# What Sexual Problems Does a Sample of LGB+ People Report Having, and How Do They Define Sexual Pleasure: A Qualitative Study to Inform Clinical Practice

**DOI:** 10.3390/healthcare11212856

**Published:** 2023-10-30

**Authors:** Andreia A. Manão, Edna Martins, Patrícia M. Pascoal

**Affiliations:** 1 HEI-Lab: Digital Human-Environment Interaction Labs, Lusófona University, Campo Grande 376, 1749-024 Lisboa, Portugal; andreia.manao@ulusofona.pt (A.A.M.); edna.m.martiins@gmail.com (E.M.); 2 Sociedade Portuguesa de Sexologia Clínica, Rua 1° de Maio No. 2, 5300-236 Bragança, Portugal

**Keywords:** LGB+, sexual pleasure, solitary sex, partnered sex, sexual problems, sexual complaints, dimensional approach to psychopathology, summative content analysis

## Abstract

**Introduction:** Sexual pleasure is a human right and a central aspect of human sexuality that contributes significantly to people’s overall well-being, making it an essential element to consider in clinical settings. This study aims to expand the understanding of sexual pleasure by examining how LGB+ people (lesbian, gay, bisexual, and other minority sexual orientations)-who perceived having a sexual problem-define solitary and partnered sexual pleasure. **Methods:** A cross-sectional exploratory qualitative study was conducted online. The current study included 85 people who self-identified as LGB+ and reported experiencing a sexual problem. Data analysis was performed using summative content analysis. **Results:** The results for solitary sexual pleasure comprised the creation of 5 categories (Enhancing the relationship with oneself, Specification of solitary pleasure, Negative experience, Unrestrained experience and A goal). For partnered sexual pleasure, 9 categories were created (The perks of being with another, Openness to experience, A result of sexual techniques, Psychophysiological experience, Misconceptions about sexual pleasure, Absence of intrapersonal constraints, Undesirable feelings, Explicit consent, and Absence of interpersonal constraints). **Discussion:** Despite reporting sexual problems, most participants reported having experienced sexual pleasure, and were able to define it. This study provided a deeper understanding of the perspectives on and experiences of sexuality among LGB+ people who experience sexual problems. Our findings highlight that current diagnostic criteria (e.g., DSM-5) do not seem to align with the problems reported by this sample population (the problems presented are beyond their sexual function). This reinforces the importance of viewing sexual problems from a perspective that goes beyond the categorial psychopathology model. Our study’s findings may offer valuable insights for the evaluation and treatment of sexual problems, where sexual pleasure is considered a crucial aspect of sexual well-being.

## 1. Introduction

People partake in sexual activities for various reasons, but the main motivation is to achieve sexual pleasure [1,2]. Sexual pleasure is the positive feelings that arise from sexual stimuli [3] and may result from various activities that involve sexual arousal, genital stimulation, and/or orgasm, among others. It is a fundamental aspect to consider in clinical work since sexual dysfunctions are “typically characterized by a clinically significant disturbance in a person’s level of sexual desire, ability to respond sexually, or experience of sexual pleasure” [4]. In other words, what characterizes sexual dysfunctions, in addition to problems with sexual functioning and the experience of distress, is also having compromised pleasure. Pleasure is a central aspect of sexual well-being and health that needs clinical attention. Thus, clinicians should move forward and beyond the restraints imposed by a categorical view of sexual problems, as many people struggle to find help and/ or interventions for their problems. For example, the categorical view may not account for human diversity, subjectivity and individual variation and could exclude people whose experiences do not fit neatly into existing categories. Plus, it may not adequately address or account for social and cultural factors that impact sexual health. Some people do not fulfil the diagnosis requirements but still experience distress and problems that impair their lives. Just because there are not enough criteria for diagnosis, these problems and suffering often are discarded [5]. Additionally, research has shown that many people perceive having sexual problems, but not all seek professional help. Some have been diagnosed, while others have subclinical complaints (i.e., do not have sufficient criteria for a diagnosis) (e.g., [6]). For example, a study found that despite effective treatments for women’s sexual problems, over half of the women with sexual problems do not seek help [7]. One reason for this may include the severity of symptoms that they experience, but other motives may be related to shame or even financial constraints. These data highlight that it is important to note that sexual problems cannot be fully understood by simply researching and studying the experiences that can be accounted for and framed by manuals or guidelines based on a categorical approach to problems. A processual view can help in understanding the manuals and guidelines better. For example, social processes play a crucial role in understanding the Minority Stress Model, which examines how minorities face social stressors related to their identity, such as discrimination and prejudice [8].

Sexual pleasure has been gaining attention lately, mainly since it is considered a basic sexual right [9]. Several researchers have also emphasized the importance of defining it more comprehensively [10,11]. According to a recent conceptual framework [10], sexual pleasure can be either a state and/or a trait. A state implies that a certain set of conditions must be met at a given moment to achieve pleasure (e.g., pleasure associated with specificities related to individual characteristics such as enjoying foreplay), while a trait is a predisposition or tendency to seek and the capacity to enjoy sexual pleasure. Both types are situational responses and differ in the cause that leads to sexual pleasure. That framework [10] emphasized that sexual pleasure is a complex experience that involves much more than just arousal and orgasm. Furthermore, there are various rewards that people can experience during sexual activity, which should be considered when defining sexual pleasure. By defining it more comprehensively, the authors highlighted that we can better capture the complexity of these experiences. It is also important to acknowledge that people can experience sexual pleasure differently, which can be influenced by cultural norms [10]. A more comprehensive definition of sexual pleasure will allow us to account for these variations and understand how cultural context might shape it. A comprehensive definition can also serve as a foundation for clinicians to understand their patients’ sexual experiences better, and it may provide a framework for developing evidence-based recommendations for clinical practice, which can help people achieve more satisfying sexual lives.

Current definitions of sexual pleasure often include solitary and partnered sex [2]. On one hand, solitary sex is characterized by engaging in sexual activities alone (such as masturbation, fantasizing, and using erotic devices, that is, visualizing photos and/or videos containing sexual activity). On the other hand, partnered sex is characterized by engaging in sexual activities with others (such as giving or receiving oral sex) [2]. Although solitary and partnered sex are commonly understood as diverse expressions of the single underlying phenomenon of sexual pleasure, their uniqueness has not been completely understood [12]. Qualitative research has revealed nuances underlining their distinctiveness, highlighting that sexual pleasure may not be a global concept without contextual specificities [13]. For example, researchers showed that solitary and partnered sexual pleasure are assumed to be oriented around the same goal: reaching orgasm [14]. However, some studies suggest that while orgasms could contribute to pleasure, people do not necessarily equate pleasure with orgasms [15]. Therefore, a closer examination of pleasure characteristics within each context is necessary to expand knowledge about sexual pleasure and its distinctiveness from other close concepts, such as sexual satisfaction and orgasms.

Historically, engaging in solitary sexual experiences, commonly designated as “masturbation”, has been associated with negative connotations, immoral behavior, and illness [16]. Nowadays, it is widely recognized as a common and beneficial sexual practice [17]. In a study conducted in a sample of 2068 women from the USA and Hungary, with a mean age of 28.8 (*SD* = 9.9; range 18–95), 94% reported masturbating in the last 12–24 months, with a medium of once per week or every two weeks [18]. These findings are partially supported by another recent study with 3878 participants from the U.S. with a mean age of 48.1 years (*SD* = 18.3, range 18–93) [17] that showed that significantly more men (60%) reported masturbating in the past month when compared to women (36.5%). Both men and women reported a patterned reason for solitary sex: to alleviate sexual tension [19,20].

Studies have shown that there are differences in the outcomes of solitary and partnered sexual activities, particularly in terms of their impact on relationships. Partnered activities tend to bring more benefits for those in a relationship, such as increased emotional intimacy [17,21]. On the other hand, solitary sex may have an indirect negative impact on some aspects of a relationship, as it is often associated with increased feelings of autonomy [13]. It may be that the interpersonal context in which sexual activity occurs directs to different goals but also to different processes and specificities in the experience of sexual pleasure, making solitary and partnered sexual pleasure two interrelated constructs worth looking at differentially. Research about the definitions of sexual pleasure developed with black women supports this assumption by emphasizing that, in a sample composed of heterosexual and LGB+ (lesbian, gay, bisexual, and other minority sexual orientations) black women, one of the four facilitators of peak pleasure is partnered interaction [22]. The experience of sexual pleasure is crucial for overall well-being, encompassing mental, physical, and interpersonal aspects. However, several factors can compromise this experience. There exists a direct and reciprocal relationship between diminished pleasure and sexual problems. On one hand, reduced pleasure serves as a significant diagnostic factor for identifying sexual problems [22]. Conversely, the presence of sexual problems can also diminish one’s overall sexual pleasure [23]. This bidirectional relationship underscores the inseparable nature of sexual problems and the experience of sexual pleasure.

Research on sexual problems and sexual pleasure has been dominated by studies examining cis heterosexual people. This set of research is usually framed by an approach that focuses on sexual function problems, which reinforces current manuals built on a categorical approach to illness and health conditions. This approach has shown that a common sexual problem for men and women is a lack of interest in sexual activity [24]. Men also reported experiencing premature orgasms [25], erectile dysfunction [26], and delayed ejaculation [27], among other problems. Women’s sexual problems often include difficulty achieving orgasm [28], not enjoying sex [29], vaginal dryness [30], and physical pain during intercourse [31]. Few studies have expanded knowledge in this field by integrating diverse samples. In a study by Peixoto and Lopes [32], with heterosexual and LGB+ ciswomen with and without sexual problems, the results showed that sexual problems may affect a woman’s desire for their partner. However, their fantasies and attraction to others remain unaffected even in the presence of sexual problems, which may suggest that sexual problems have a more partner-specific and contextual impact. It is important to note that this quantitative study did not explore the differences and similarities across sexual orientations. Additionally, considering that all research has some limitations, the results of that study may have been influenced by using measures designed for heterosexual people and by approaching sexual dysfunction based on a categorical vision sustained in the measures used to assess it.

Our literature review found few studies conducted on the definitions of sexual pleasure in LGB+ people. In one study, the sample consisted of heterosexual and LGB+ black women who reported positive feelings about sexual pleasure and defined it in hedonic (pleasure based on feelings and sensations) and eudemonic (pleasure based on goal attainment) ways [22]. Another study carried out with gay men in Mexico [33] suggested that gay men described a unique form of masculinity as highly regarded within erotic contexts, highlighting that the masculinity of the partner was desired as a form of fetishization. A study of young students who self-identified as gay men students explored the meanings attached to gay sexuality through self-labelling practices [34]. That study revealed that bottom identity (i.e., A “top” is defined as someone who prefers the insertive role and a “bottom” as someone who prefers the receptive role in penetrative sex) may be indicative of a self-serving bias to maintain a positive self-identity. However, the authors also emphasized the importance of understanding how and why young men identify with power and vulnerability beyond stereotypes. Even though explored the meaning of sexual pleasure, the study does not provide us with any explicit definition of this construct. Another study included a sample of heterosexual women and women who have sex with women [13]. Based on data collected with cis women, the study suggested that sexual pleasure is a concept that, although there are also some similarities between the two groups of women, may be defined differently by LGB+ people compared to heterosexual peers. For example, women who have sex with women expressed more ambivalence regarding solitary masturbation and partnered orgasm. This finding about the differences between sexual orientations is consistent with previous research on LGB+ experiences (e.g., sexual satisfaction) that demonstrated that heterosexual people and LGB+ people’s experiences differ, as LGB+ people present experiences marked by issues related to concealing sexual identity development and feelings of pressure towards heteronormative experiences [15,35]. Although some quantitative [36] and mixed methods studies have highlighted the gendered nature of sexual pleasure and how dominant heteronormative scripts frame sexual pleasure, there is still much to be explored. For example, research on transgender people is still framed by heteronormative scripts [37].

Laan and colleague’s [38] reviewed biopsychosocial evidence on the gendered ability to experience sexual pleasure and found that sexual pleasure remains an unexplored construct, especially in marginalized populations, such as LGB+ people. The authors highlight that although some studies have examined sexual pleasure in this population, most of the sex research has focused on cisgender, heterosexual, Western, educated, industrialized, rich, and democratic (WEIRD; [39]) contexts, and most of them used quantitative analysis. This suggests that definitions of sexual pleasure often fail to encompass the perspectives of people who do not identify as cisgender, heterosexual, or belonging to WEIRD contexts, and it supports Werner and colleague’s [10] claim that academics need to define sexual pleasure better and consider different contexts of the experience of sexual pleasure. Quantitative studies about people who identify as LGB+ are also lacking. A recent scoping review on psychosocial and behavioral aspects of women’s sexual pleasure [40], which analyzed data from 76 studies, found that only two studies included samples of lesbian and bisexual women.

While research suggests that LGB+ people may face stressors leading to unfavorable health outcomes [41], sexual dysfunctions [42,43], and delay in seeking professional help [44], it is crucial to gain insight into their experiences to improve healthcare access.

To our knowledge, no studies have explored how the experiences of sexual pleasure of LGB+ people who perceived having sexual problems are defined, and this is also due to the fewer opportunities given to this population to define and report their own experiences. In line with this, the current study aimed to expand knowledge about sexual pleasure experiences by delving into definitions of sexual pleasure, both solitary and partnered, among LGB+ people who perceive having a sexual problem. By shedding light on how this population defines sexual pleasure, this research offers insights into the complexities of this construct, clarifying the interconnection of the experience of sexual problems and the meanings of pleasure, ultimately contributing to a more inclusive understanding of human sexuality. As an exploratory qualitative study, we did not have a prior hypothesis. Instead, we posed a broad research question (“How do LGB+ people who perceived having a sexual problem define their experience of sexual pleasure?)”.

## 2. Materials and Methods

### 2.1. Research Design

This study is part of a larger cross-sectional online project on the correlations and meanings of sexual pleasure, a collaboration between the Sex Education Museum (MUSEX), the Master’s Degree in Sexology at Universidade Lusófona and the Portuguese Society of Clinical Sexology (SPSC). We developed an anonymous online cross-sectional study that collected quantitative and qualitative data from a convenience sample. The organizations’ representatives reviewed the survey protocol to ensure it was adequately relevant, used inclusive language, and was not too long. People who identified as LGB+ also reviewed the content and language of the study and tested its inclusiveness and adequacy. After reaching a consensus, the final version was implemented online using a secure platform (Qualtrics). The data used in this study consisted of three parts. The first part contained the informed consent, and the second part contained the socio-demographic data (such as age, gender, sexual orientation, and relationship configuration). The relationship configuration was categorized into “Without romantic relationship/s”, “Monogamic”, “Consensual Non-Monogamic”, “Sporadic Relationships”, and “Another option” (e.g., having a relationship with another person but without future common plans). The third part gathered qualitative data. First, people were asked if they had any sexual problem(s). If so, people were asked to describe their sexual problem(s). Participants were then asked two questions about the meaning of sexual pleasure (solitary and partnered).

Based on the literature review, this specific study used the data that aimed to explore the definitions of solitary sexual pleasure and partnered solitary sexual pleasure among LGB+ people who perceived having a sexual problem as described by LGB+ people. To identify a sexual problem, participants were asked: “Do you think you have a distressful sexual problem? If yes, could you explain what the sexual problem is?”. People’s descriptions of sexual problems were collected. Before collecting definitions of solitary and partnered sexual pleasure, the constructs of solitary and partnered sexual experience were presented. Then, to ensure people could deliver informative answers, we presented examples unrelated to the study’s themes of non-informative and informative qualitative answers. After that, people were invited to present a definition of sexual pleasure according to their experience in the two contexts (partnered and solitary); two open questions were asked: “Based on your personal experience, what is solitary sexual pleasure?” and “Based on your personal experience, what is partnered sexual pleasure?”.

### 2.2. Ethical Consideration

This study obtained ethical and deontological approval from the host institution. The authors followed the principles and guidelines in the Helsinki Declaration and the European Textbook on Ethics and Research [45,46]. Informed consent was obtained from all participants, a contact was provided in case participants had questions, and a list of resources for sexual health was presented. No IP was saved, and geolocation information was deleted. A password protects the database; only the research team can access the information collected.

### 2.3. Participants

A non-probabilistic sample of 85 LGB+ people who self-identified as having a sexual problem was included in the study. The participants’ age ranged from 18 to 63 (*M* = 32.49, *SD* = 11.44). Regarding sexual orientation, 6 people identified as “Lesbian women” (7.1%), 14 as “Gay men” (16.5%), 32 as “Bisexual person” (37.6%), 7 as “Queer person” (8.2%), 13 as “Without category/label” (15.3%), 4 as “Questioning” (4.7%), and 9 as “Another identity not mentioned” (10.6%). Regarding gender, 50 participants self-identified as “Women” (58.8%), 26 self-identified as “Men” (30.6%), 5 as “Non-binary person” (5.9%), 1 indicated “I do not know” (1.2%), 2 as “I prefer not answering” (2.4%), and 1 person as “Another identity not mentioned” (1.2%). According to the American Psychological Association [47] guidelines, the survey always includes options for “Other” and “Prefer not to say” for gender-related variables. When asked about the participants’ relational configuration, 20 had “No significant relationship/s” (23.50%), 39 were in a “Monogamous relationship” (45.88%), 14 were in a “Consensual non-monogamous relationship” (16.47%), 10 had “Sporadic relationships” (11.76%), and 2 identified with “Another option” (2.35%). All participants reported perceiving that they have a sexual problem. Problems reported by participants underwent a summative content analysis [48] (codification).

### 2.4. Data Collection Methods

Online data were collected from January until March 2023 after advertising the URL through social networks and using a snowball technique, i.e., people in social networks were invited to disseminate the survey throughout their social media and personal contacts. Participants in the study were not offered any incentives and could stop their participation without any consequences. It is impossible to determine the compliance rate of the study, as it was advertised through online newsletters, and it is unclear how many people were reached and read them. The inclusion criteria for the study were: (1) being over 18 years of age (age of consent), (2) mastering the Portuguese language, and (3) having had sexual interaction (which means mutual stimulation of genitals, oral sex, and other forms of sexual stimulation [49]). For the current study, we used data from 85 people who self-identified as LGB+ and who perceived having a sexual problem. Among these, 80 people described their sexual problems (see Section 3).

### 2.5. Data Analysis

The methodology used for data analysis was the summative content analysis [48,50]. Two researchers, A.A.M. and P.M.P., independently and simultaneously coded the data. Afterwards, they discussed it until a consensus was reached. More specifically, the first author (who has both clinical and voluntary experience working with LGB+ people) and the third author (a sexologist, psychotherapist, and cognitive-behavioral supervisor who also has performed clinical work with LGB+ people) analyzed the responses. They followed a specific procedure that included (1) reading the transcripts multiple times to gain familiarity with the content, (2) defining the unit of analysis for the transcriptions, (3) creating a coding schema by analyzing the transcriptions, (4) testing the coding scheme for consistency and clarity on a sample of transcripts to ensure high levels of consistency, (5) categorizing all the transcriptions, and (6) rechecking the coding consistency to ensure reliability. At first, the codes were semantic. Later, the responses became more detailed, precise, and aggregated around a common latent meaning.

We created categories for the study material using an inductive method [50]. This data-driven approach allowed us to explore the topic freely without being confined to structured theories.

We did not use any software to manage the qualitative data. Quantitative data, which is merely descriptive, was handled using IBM SPSS, Version 26 [51].

Categories are presented in bold, codes are underlined, and quotes are presented in italics.

## 3. Results

Participants’ responses regarding sexual problems (Table 1) were usually very short (e.g., *Lack of interest in sex*) and sometimes focused on sensitive topics (e.g., *Betrayal on my part and [I am attracted] to someone of the opposite gender to my partner’s* or *I suffered of sexual abuse*). Still, they were diverse, with patterns found considering sexual orientation and gender. The difficulties in anal sex (being active) and reconciling the desire for people of the same gender were reported by men who have sex with men. Additionally, problems with sexual pain were most reported by men. Women particularly reported problems related to lack of libido. Problems such as being in a monogamous relationship with a person of another gender and having a desire to have sex with people of the same gender were reported by bisexual people.

Of the 85 participants who were perceived to have a sexual problem, 80 described their sexual problem/s. Among these, 21 reported more than one sexual problem (26.3%), 17 reported having two sexual problems, 3 reported having two sexual problems, and 1 participant reported having four sexual problems. No pattern was found between the quantity of perceived sexual problem/s and the sexual problem/s.

The results are presented in the same order as the questions were asked. Problems reported by participants are reported in Table 1.

Regarding the definitions of sexual pleasure, participants provided direct and reflexive answers, often including examples of their sexual experiences and desires, probably prompted by the instructions that directly appealed to the disclosure of definitions based on people’s experiences.

There was no discernible pattern between the length of responses and the age or type of sexual problems of the respondents.

Similarly, no pattern was found between those attracted to just one gender (e.g., lesbian women) and those attracted to more than one gender (e.g., bisexual men) regarding their definitions of sexual pleasure.

Categories and code frequencies are shown in Table 2 and Table 3.

### 3.1. Based on Your Personal Experience, What Is Solitary Sexual Pleasure?

The responses received from the participants regarding the definitions of solitary sexual pleasure were informative and varied in length, ranging from medium-length responses (two or three sentences) to extensive sentences (more than four sentences). Examples can be seen below, ad verbatim.

From the identification of the codes related to the definition of solitary sexual pleasure, we established five categories, namely, **Enhancing the relationship with oneself**, **Specification of pleasure**, **Negative experience**, **Unrestrained experience,** and **A goal**.

In the category **Enhancing the relationship with oneself**, participants explained that solitary sexual pleasure is a way of getting to know themselves better sexually, thus leading to the identification of the Self sexual knowledge code. Examples of this code are: *It is the time when I get along with myself the most, and I often get to know myself* and *Self-knowledge and also the possibility of knowing and exploring personal limits*. Furthermore, participants reported that solitary sexual practices are often used as a strategy, almost a ritual, to reduce negative emotions and increase positive emotional experiences. In this way, we defined the code Positive mood inducer: *sexual pleasure resulting from masturbation is, for me, something that helps to relieve stress or to get out of a state of apathy* and *for me, it has always been a way of relaxation, where I touch myself as I like best and whose ejaculation helps me to release stress*. Another code was the Promotion of emotional well-being, for example: *The ability to imagine or recall previous situations in thought produces happiness, affection, […] emotional well-being*. We also identified another code called the Promotion of physical well-being. Here, participants emphasized that *Sexual pleasure […] is good for health, [for the] physical [health*].

Another identified category was the **Specification of solitary pleasure**. Here, we pointed out three codes, namely, Strictly self-orgasm-oriented, More exploration of sex toys, and Focus on imagination. In the Strictly self-orgasm-oriented code, the participants highlighted that solitary sexual pleasure is a type of practice in which they focus on achieving orgasm: *In masturbation, orgasm is always reached, as opposed to what happens if I’m with someone else. And it is this pleasure that I’m looking for and that I know I can find in masturbation.* In the More exploration of sex toys code, participants mentioned that one of the specificities of this practice is a total freedom using sexual toys to enhance the pleasure felt: *Lately, I have been experimenting with using a chastity belt with an anal plug to feel more pleasure. After a few hours with the chastity belt on and when I watch pornography or have sexual conversations with friends, I take it off and masturbate, with a more intense result* and “I *use sex toys (mainly clitoral suckers*). The code Focus on imagination highlights the use power of imagination in the solitary practice: *I use pornography to masturbate, and I only feel more pleasure, when watching pornography, I imagine myself in the place of the person who is penetrated, or I imagine myself as a voyeur, commenting on what I see and giving recommendations. In these cases, masturbation becomes more intense, and I feel greater pleasure*. Lastly, the participants also emphasized that solitary sexual pleasure is a form of Focus on self-pleasure, stating that: *Giving pleasure to oneself is something that encompasses independence is […] a moment that we have so intimacy with ourselves, as we want, in our way, with the sole purpose of giving ourselves pleasure, holistic* and *focus only on me. Enjoyment and satisfaction are guided by me*.

In the category **Negative experience**, participants mentioned negative aspects of the practice of solitary pleasure, namely, Lack of fulfillment, Negative feelings, and Routine. Participants indicated that solitary sexual pleasure gives them a feeling of not reaching a peak of pleasure, a code that we call Lack of fulfillment. That is, *alone I can never reach climax because the sensation is too much for me, so I can not reach the end, and I have to stop* and *it is good, but it is quite quick and easy to achieve [orgasm], so it ends up being boring*. Participants also highlighted that it is easier to reach orgasm through this solitary practice, although it is less pleasurable. For example: *Having more control over action, I can achieve orgasm more easily, but they always feel much less like a moment of full-body explosion and just like a localized sensory peak, which makes the experience sometimes frustrating*. Within the unpleasant feelings (Negative feelings), the participants reported about solitary sexual pleasure, stating that: *It is something that I rarely do, or rather, that I try not to do. In the end, I just end up feeling frustrated*, *Sometimes embarrassing, because I still feel that I am doing something sinful*, *Masturbation is something that brings me shame, and when I sometimes feel the need to touch myself, sometimes it is frustrating* and *Masturbation has always been complicated for me. As a trans man, it began as a source of dysphoria*. Solitary sexual pleasure was also characterized as an experience that increased the feeling of isolation: *Since I was in my 40s, it started to make me feel more isolated because there is no shared intimacy and I do not have a partner*. Lastly, participants mentioned that it is a less exciting practice since *[I masturbate very often, almost as an addiction. I masturbate more than 3 times a day, every day]. For this reason, I feel that I no longer enjoy masturbating. I do it because it has already become a kind of routine*, so we identified another code called Routine.

Regarding the category **Unrestrained experience**, we identified three codes, namely, Liberation, Sensorial experience, and Letting oneself go. The Liberation code focused on the freedom that participants indicated they felt: *It is a form of liberation* and *When I masturbate, I just want to reach the height of the issue.* The Sensorial experience code reinforced that this practice allows a person to explore all sensory sensations, for example, *The pleasure of masturbation is an explosion of sensations. Stimulation of the penis turns it into an organ where each area causes and transmits different sensations. Stimulating with the whole hand or with the tips of the fingers induces different pleasures. Touching the glans or the base of the penis causes goosebumps of different characteristics. The act of ejaculating creates a chilling spasm in the body that elevates the pleasure to the highest level*. Considering the Letting oneself go alone code, the participants mentioned that the solitary sexual act allows them to lose control, for example: *Feeling the lack of control growing. Feeling that I lose conscious control of the body and sensations that emanate from it*.

**A goal** was another category identified, where participants listed some uses of this sexual practice. The usefulness of solitary sexual pleasure is related to it being a practice that people can carry out even in the absence of a sexual partner, making it a code identified with Pleasure even in the absence of another, for example: “Giving ourselves pleasure when we do not have someone to share it with at the moment” or “Masturbation is a practice that helps me when I do not have sex for more than a week. I like touching myself, I like to enjoy, but I prefer to do that with other people”. Another code identified was A path to partnered sex, where participants emphasized that they used these solitary practices before being with their partner to enjoy sexual practices with their partner better. For example, *I also tend to masturbate when I know I’m going to be with my boyfriend because I get more excited,* and *I can derive pleasure from masturbation because it stimulates the sexual act that may arise*. The last code identified was Solitary sex is more pleasurable than partnered sex, where participants compared the pleasure felt in solitary sex and partnered sex. Examples of this category were: *I feel much more pleasure in masturbation than in sexual intercourse with another person. Because I feel my body and I know what I like* and *Sexual pleasure while masturbating is purely physical, and more explosive compared to when it is with a partner.*

### 3.2. Based on Your Personal Experience, What Is Partnered Sexual Pleasure?

Participants provided a wide range of responses when asked what they thought partnered sexual pleasure meant. In the same way as solitary sexual pleasure, some participants gave medium-length answers (two or three sentences), while others provided more detailed responses (more than four sentences). Here are some examples, ad verbatim.

From the identification of the codes, one of the categories is defined as **The perks of being with another**. This category details participants’ opinions regarding the huge importance they give to having a partner to fulfill a positive sexual experience. One of the identified codes was named Interconnection of emotions and physical experience, which focused on the emotional and physical connection with the partner, for example, *Having sexual pleasure means fully enjoying the union with my partner, being able to physically and emotionally feel every sensation of that moment, freely* and *It is a feeling of surrender to the* other. Honest communication was another code pointed out, where positive aspects of good communication between partners were emphasized: *clear and honest communication*. Safe place was another code, namely, *to create a safe space where everyone involved feels comfortable*. We also created a code named Power dynamics, for example, *I consider it essential to have a lot of sensory stimulation, the exploration of fetishes and some dominator/subjugated dynamics to feel pleasure*. Another code identified was The pleasure of pleasuring, where participants reinforced the pleasure it gives them to pleasure another person: *My sexual pleasure comes mainly from recognizing how the other person feels and how I am making them feel. I like to feel that there is mutual pleasure. It is one of the things I look for the most*.

Another category was created, namely, **Openness to experience**. One of the codes was Letting oneself go with a partner (e.g., Sometimes it reaches such an intensity that it is as if I lose control over my own body, and I let the feeling of desire guide my movements, not thinking about the act itself. At these moments, the heat spreads over the whole body, and I get completely goosebumps and have slight dizziness and vision loss). Another code was called Exploration of the five senses, in which participants emphasized the pleasure they feel when exploring the body’s five senses, for example, it is about exploration, discovery, touch, context, words, and all the enveloping features of the act, and they do not necessarily have to go through explicit things. Soft touches, massages, blowing, certain environments, sounds, odors, flavors, all the activity that stimulates the libido arises from the set of senses. Take tantric sex as an example. It goes through several stages. Furthermore, we defined the code Being in the present moment (e.g., when we feel so much that we seem to forget everything else around us and enjoy the present moment. a moment when everything else ceases to exist).

Considering the category, **A result of sexual techniques**, five codes were highlighted, namely, BDSM/FET (BDSM = Bondage, Discipline, Dominance, Submission, and Sadomasochism; FET = Fetish), Oral sex, Anal sex, Finger penetration, and Mutual masturbation. The code BDSM/FET (e.g., *Feeling that another man has some dominance over me is a preponderant factor of sexual pleasure—submission, in this case*) demonstrates specific forms of sex that participants feel they enjoy. Furthermore, we defined the codes Oral sex (e.g., *I like oral sex*), Anal sex (e.g., *I have sexual pleasure with anal sex*), Finger penetration (*[I like] finger penetration*), and Mutual masturbation (*[I like] mutual masturbation*) that demonstrates types of sexual techniques that the participants emphasized they liked.

Another category identified is called **Psychophysiological experience**. Two codes were recognized in this category: Intense desire/arousal and Dependent on orgasm. Regarding the code Intense desire/arousal, answers focused on the pleasure that the participants refer to when feeling desired and when desiring their partners: *Feeling desired, that is, realizing that the person finds me attractive or beautiful, also gives me a lot of pleasure* and *It is hard to explain, but sexual pleasure may not involve sex, sometimes just talking, the imagination, is enough to leave that feeling of almost euphoria, of wanting to feel the softness of the skin, the warmth of being skin to skin.* In the code Dependent on orgasm, the participants demonstrated that one of the characteristics they identify in partnered sexual pleasure is the focus on orgasm. In other words, for these people, partnered sexual pleasure seems to translate into orgasm. For example: *Sexual pleasure it is to reach the highest point*.

Regarding the category **Misconceptions about sexual pleasure**, we identified a code where participants mentioned that partnered sexual pleasure does not necessarily have to culminate in orgasm, which we named It does not depend on orgasm. For example, *Pleasure does not stop at orgasm* and *Pleasure independent of orgasm*. Furthermore, another code was identified: It does not depend on penetration. Some examples are *Sexual pleasure with the various male and female partners was very fluctuating. I feel that I have never had an experience with much sexual pleasure with men because sexual pleasure is very directed towards penetration, and I have vaginismus* and *Sexual pleasure for me is not just related to the act of penetration or stimulation of the sexual organs, but the whole environment as well. The famous quickie does not represent pleasure; the way to reach the climax contains much more than penetration*.

Participants reported aspects related to them that prevented them from feeling sexual pleasure, categorized as the **Absence of intrapersonal constraints**. This category contains two codes, namely, Physical illness and medication (e.g., *Physical and mental health problems and the respective medication kept me away from sex*) and Negative states and emotions (e.g., *I can feel sexual pleasure if I am stimulated, not tired and/or having personal problems at the moment*).

We defined a category named **Undesirable feelings**, in which participants mentioned negative aspects of having pleasure in sex with another person. Here, participants highlighted that partnered sex is a Difficulty in having pleasure, that is, “*For me to have pleasure, I can only truly do it alone” and “I can no longer feel pleasure from it [partnered sex]”*. Partnered sex was also characterized by a code we named Trigger of insecurities, in which participants mentioned that: *I’m not in good physical condition, after 5 min, I’m exhausted. Having to undress is uncomfortable, having to expose myself* and *[where do] my insecurities arise (on a physical and/or relationship level)*. They mentioned that partnered sex is *a waste of time from my perspective*, a code that we named Waste of Time. Lastly, the participants mentioned that they have difficulty giving themselves to others, and we created a code named Difficulty in surrendering to others: I *can never surrender to the point of feeling pleasure*.

Another category was called **Explicit Consent**, in which the participants affirmed the importance of having explicit consent to receive and give partnered sexual pleasure, which we named Consent. Some examples are: *Sexual pleasure for me comes from a mutually consenting* and *[sexual pleasure] it is not limited only to the sexual act, but also to […] consent*.

Lastly, we also defined a category named **Absence of interpersonal constraints** to represent relational aspects that participants demonstrated that prevent them from feeling sexual pleasure. We identified three codes. The first we named Comment about the body (*To obtain sexual pleasure—something difficult for me—it is essential that the person does not have certain red flags such as derogatory comments about my body*). The second we called Comparison with ex-partners in which participants reenforced that if the partner comes with a comparison to an ex, the pleasure is over (*The comparison, such as: My ex liked it*).

## 4. Discussion

The current study used a qualitative cross-sectional online methodology to gather information from LGB+ people who perceive themselves to have sexual problems and, namely, how they describe their problems and how they define sexual pleasure (both solitary and partnered sexual pleasure).

Regarding the description of sexual problems, it is not possible to know if the participants met the criteria for the diagnosis of sexual dysfunctions. However, we wanted to focus on the perception of having a distressful problem independently of the existence of a formal diagnosis. Hence, the study was informative as it emphasized that the participants’ problems are beyond their sexual function and take a broader perspective on the themes that are a focus of distress (e.g., DSM-5 [4]).

In line with other research in the field, the answers to what constitutes a sexual problem highlight experiences related to intrapersonal difficulties, personal trauma, and interpersonal problems. While some of these issues may be experienced by heterosexual people too (e.g., trauma caused by sexual abuse and the experience of sexually compulsive behavior [4,52]), others may be more frequent in LGB+ people (e.g., men’s difficulties with being receptive to anal sex), and others are specific to sexually diverse samples (e.g., bisexual participants report concealing their same-gender sexual desire from their different-gender partners as a sexual problem). These results suggest that this is an unexplored area, and the specificities of sexual distressful experiences in LGB+ samples need further investigation to better inform clinicians about possible paths of inquiry but also to work close to LGB+ samples to better advocate for the promotion of sexual health and implement suitable and effective interventions.

Regarding sexual problems, participants perceived the absence of a partner as a barrier, even though they experienced sexual desire and arousal. This result seems to be related to a recurring problem found in sexology practice, which is the discrepancy in desire [53]. This may be because it may be more difficult for LGB+ people to find partners. Other interpersonal factors, such as another person’s lack of sexual desire leading to abstaining from sex or the routine in the relationship, were also highlighted as inhibitors of sexual desire. These results align with the concept of sexual inhibition of the Dual Control Model [54], which suggests that people possess two propensities, sexual excitation and sexual inhibition, that impact their sexual responses [55]. Although some levels of sexual excitation and inhibition are associated with healthy sexual functioning, high sexual inhibition and low sexual excitation are associated with a greater susceptibility to sexual problems [56]. Studies have confirmed the existence of these propensities across various sexual orientations. For example, in a study with homosexual and bisexual women [57], the results showed that the relationships between sexual excitation, inhibition, and sexual function in LGB+ women may be similar to those observed in heterosexual women.

Lack of sexual desire and having sexual pain were predominant sexual problems in our sample. Problems related to lack of sexual desire and sexual pain can be understood within the framework of the classic sexual response model. The traditional model of the human sex response cycle includes desire, arousal, orgasm, and resolution [14,58]. Lack of sexual desire can be associated with difficulties with desire. In other words, if a person does not experience psychological or physiological arousal, then it can lead to a lack of desire for sexual activity. Sexual pain can be caused by a variety of factors that disrupt the normal progression of the sexual response cycle, such as lack of lubrication, medication and health conditions. It is typically experienced during the sexual response cycle, often during or after the arousal phase. This way, a lack of sexual desire and sexual pain can be understood along the lines of the classical sexual response and psychopathology manuals (e.g., DSM-5; [4]).

However, some content in the reported sexual problems (e.g., having dissatisfaction with relationship configuration or not feeling sexual attraction at all) was more compatible with views beyond psychopathology manuals. For example, a different approach to addressing sexual problems, known as the “New View”, was developed as an alternative to the categorial psychopathology model as a comprehensive framework to understand women’s sexual problems [59,60]. This new perspective emerged in 2000 after recognizing several problems related to women’s sexuality through a biomedical lens. One major critique of this model compared to the categorical psychopathology model is its failure to account for the broader context of relationships when considering sexual issues [59,60]. Instead, the categorical psychopathology model tends to focus on purely physical and physiological aspects, often neglecting other important factors. Even though the DSM-5 has proven to be an asset, as it integrates a dimensional view into diagnosis [4], there are still some problems with its categorical approach. For instance, people who identify as part of marginalized populations, such as those who self-identify as LGB+, may encounter obstacles in expressing their specific characteristics and capabilities [61]. In this way, we assume that some underlying aspects of sexual problems (even if directed towards physiological difficulties) must be considered within the context of a holistic approach and framed by its differentiator elements (e.g., being part of the LGB+ population).

Considering the social-based Minority Stress Model [8], LGB+ people experience several chronic stressors ranging from external, objective, and prejudice events, such as discrimination and violence (distal stressors), to internal, subjective events stemming from these external stressors, such as isolation and fear of rejection (proximal stressors). These stressors can have adverse effects on mental health [62] and sexual well-being [63]. Due to the minority stressors, LGB+ people may have fewer opportunities to have relationships with other people, just as daily microaggressions may impact their sex health. For example, if a person often feels discriminated against, they can isolate themselves to prevent more discrimination episodes and, due to this, not have a romantic or sexual relationship. In fact, some of the participants in the current study mentioned that not having a sexual partner is, for them, experienced as a sexual problem. Additionally, LGB+ people face other challenges, such as limited access to healthcare services [64], which may also contribute to inhibitions in sexual expression.

Other factors also emerged, such as lack of confidence, assertiveness, and the presence of sexual anxiety. It is crucial to note that, although these last factors are not unique to LGB+ people, they face additional challenges due to minority stressors within a heteronormative and heterosexist culture that may promote them. In fact, research has shown that LGB+ people’s sexual problems may depend on their specific sexual expressions and biopsychosocial factors, which may mean that clinical sessions should contemplate a constellation of factors within the specificities of these people (e.g., negative sexual beliefs and attitudes towards themselves, not accepting their own’s fantasies [65]).

Some of the participants also indicated that they felt sexual compulsivity was a sexual problem (e.g., *excessive masturbation*). According to a study [66], engaging in compulsive sexual behaviours may be a way to regulate emotions. In fact, research by Pachankis and colleagues [67] with gay and bisexual men showed that internalized stigma, or homonegativity, is associated with sexual compulsivity. This internalized stigma can be seen as a form of shame related to having a minority sexual identity. Recognizing that social and cultural challenges may limit the sexual experiences of LGB+ people (e.g., *pleasure is difficult for me*), it is essential to view sexuality from a perspective that goes beyond the categorial psychopathology model. This means considering the diversity of possible experiences and not disregarding them.

Furthermore, our results indicate the existence of comorbidity among sexual dysfunctions (since 21 participants reported having distress from more than one sexual problem), thus highlighting the importance of following a transdiagnostic approach and implementing treatments accordingly. Literature suggests that targeting transdiagnostic factors, rather than individual symptoms, can lead to more effective and efficient treatments. A dimensional approach can intervene in a wider range of problems [68].

Our results regarding the sexual problems revealed that, even though the participants recognized a sexual problem in themselves, they experienced sexual pleasure and proposed to define it.

Regarding the answers to the definitions of partnered and solitary sex, taken together, the answers were informative and revealed different definitions and, therefore, distinctive features for each experience. During the study, participants shared their experiences, and findings showed that negative emotions could be associated with the experience of pleasure. This is important because sexual pleasure is usually viewed as a positive experience globally, which often overshadows these negative aspects in clinical practice. These negative emotions can discourage people from engaging in sexually pleasurable activities, leading to psychological distress. For instance, feelings of shame and guilt about having or enjoying sex can contribute to poorer emotional outcomes (e.g., anxiety and depression [69]). Additionally, LGB+ people who are in same-gender relationships and who have sexual encounters may experience fear, shame, and guilt from not conforming to normative standards of sexual orientation and sexual and romantic behaviour, including those who have disclosed their sexual orientation [70,71].

On the one hand, concerning the definitions of solitary pleasure, our participants’ answers emphasized the important role of orgasm, a well-established correlate of sexual pleasure. This may differ from heterosexual samples since other research shows that heterosexual women who have orgasms through solitary sex feel like they are stealing orgasms from sex with their partners; thus, they may not consider them as something positive [13]. At the same time, other studies showed that heterosexual men use orgasms of solitary sex to compensate for their dissatisfaction with orgasms derived from partnered sex [72]. In other words, in the heterosexual population, orgasms from solitary sex seem to be usually associated with pleasure that is not tangible through partnered sex, whereas in the LGB+ population, orgasming in solitary sex can be a source of pleasure by itself, not being perceived as something necessarily associated with partnered sex [13].

Our results support the positive role that solitary sex has. It is an opportunity for self-development, unconstrained experiences, and expansion that may optimize partnered sexual experiences [73]. Our results are in line with other research that presents solitary sex as an antidote to stress [16] that can alleviate negative emotions, such as feelings of inadequacy [74] or shame [75]. Still, although solitary sex can be used as a coping mechanism, sometimes it may be less effective in reducing negative emotions or have a double effect, reducing stress but creating more shame and guilt because of masturbation or other types of solitary sex. In other words, while solitary sex can serve as a powerful tool for stress relief and emotional well-being, it is important to recognize that it may not always be a straightforward solution. Some people might find that their solitary sexual experiences evoke a dual effect, reducing stress and triggering more intense feelings of shame and guilt. Additionally, shame is particularly important considering that research with LGB+ people shows that this shameful emotion is more frequent in this population than in heterosexual peers, e.g., [76]. This also indicates that when approaching sexual pleasure, clinicians should look into diverse intrapersonal non-sexual experiences outside of the sexual practices realm. For example, shame can be associated with one’s sexual orientation [77]. This may prevent people from exploring solitary sex’s full potential as a tool for sexual self-knowledge [73]. This paradoxical outcome may occur when internalized homonegativity and societal expectations about sexual behavior clash with one’s intimate desires and attractions.

On the other hand, partnered sexual pleasure is presented as a multisensorial intense experience linked to feeling protected and understood in an interpersonal context, which can be linked to the responsiveness detected in other people [78]. The definitions of the participants of this study are in line with a proposed framework for the comprehension of sexual pleasure [10] that accentuates the individual capacity (a trait) to do so because our participants referred to the importance of openness to experience as an essential aspect to consider the definition of pleasure, as well as the contexts in which sexual activity occurs (state). The authors also referred to positive (domain of techniques) as well as negative (lack of explicit consent) contexts that are required and framed the experience of sexual pleasure, indicating contextual changes within partnered sex, which was in line with our participant’s answers. It is worth noting that the domain of specific techniques of mutual stimulation is important, which suggests that sex education programs and interventions aimed at maximizing sexual pleasure may be an opportunity for improving partnered sex. These results are in line with previous research that has highlighted the importance of knowing the most effective ways to stimulate a partner [79] in order to have a pleasurable partnered experience.

Tautologically, definitions of partnered sex highly focus on the partner and on the pleasure of the partnered experience. When describing sexual scripts (i.e., steps towards partnered sex), the participants of this study usually referred firstly to foreplay, focusing on a sequence of interconnected behaviors involving the other’s body. One example is kisses and exploration, commonly progressing toward penetration or genital contact, thus culminating in mutual pleasure [80]. It is important to note that the pleasure our participants talked about is not a synonym of orgasm but instead a diversity of dimensions that contribute to and reflect pleasure. For example, according to participants, a higher level of sexual pleasure can be attained when establishing a deep emotional connection with the partner [81] or when effective communication is present [82].

Participants’ answers showed that when others make comments about their bodies, it hinders their ability to experience sexual pleasure. Since social pressures and beauty standards often drive body dissatisfaction [83], the impact of external commentary about the body may further increase body dissatisfaction. A recent study [84] showed that body dissatisfaction impacts sexual variables (i.e., sexual satisfaction) in LGB+ cis people, particularly in LGB+ cismen, suggesting that body dissatisfaction may be related to the perception of sexual value in LGB+ cisgender men, and may contribute to their experiences of sexual problems or pleasure.

In a comprehensive review, Lick and colleagues [85] found that LGB people face physical health disparities compared to heterosexual peers. These disparities include overall health, activity limitations, disability, disease risk factors, and cancer, and happen largely due to the stress resulting from social stigma, which is experienced throughout the lifespan [86]. This highlights, as our study, the urgent need to address not only medical care but also the social and psychological well-being of all ages LGB+ people, especially in terms of sexual well-being. Understanding how physical health disparities affect the sexual experiences and challenges of LGB+ people is a complex and important area of study.

Considering that the people who participated in this study have sexual problems, it is not clear to what extent the definitions they presented of sexual pleasure are related to the idealization of the pleasure felt before they had the problems or whether they reflect the characteristics of the pleasure they currently feel in the context of sex life with problems. The intersection of physical and mental health plays a crucial role in shaping the specificities of sexual problems. The sexual problems addressed by the participants in this sample may be a reflection of the impact that minority stressors have on the physical and psychological health of LGB+ people. Once again, this reinforces that it is essential to view sexuality from a perspective that goes beyond the categorial psychopathology model.

Clinicians working with LGB+ people should take into consideration these findings to inform their practices, advocate for sexual health promotion, and tailor interventions to meet the unique needs of this population. More specifically, clinicians should be attuned to the diversity within this population, acknowledging that people sexually attracted to different genders may find themselves in either heterosexual or same-gender relationships, even when feeling that being attracted to more than one gender causes significant distress due to societal expectations and personal desires. It should be noted that, in line with our study, the specificities of LGB+ people can translate into sexual problems that are also specific to this population. Clinicians should consider the impact of negative intrapersonal relationship, including feelings of inadequacy and shame, especially in the context of sexual orientation-related shame. When shame is associated with sexual orientation, it can become a significant barrier to exploring one’s own desires and achieving a deep understanding of sexual identity. Overcoming this shame involves not only self-acceptance but also a reevaluation of societal norms and prejudices. It is important to create a safe and non-judgmental space for LGB+ people to explore their sexuality and enjoy the benefits of solitary sex without the burden of unwarranted shame or guilt. It is also important that clinicians who work with LGB+ people recognize the need for a comprehensive approach to addressing sexual health concerns, one that acknowledges the complex interplay between sexual pleasure, psychological well-being, and the unique socio-cultural factors influencing LGB+ people, recognizing the significance of contextual factors when addressing sexual health concerns. By addressing these contextual factors, clinicians can provide more effective and culturally competent sexual patient care. Lastly, clinicians should also be aware that the experience of sexual pleasure can lead to negative emotions and processes and the impact that this has, and they should explain the rationale of these processes to the client.

### 4.1. Limits

The lack of face-to-face contexts where meanings could be deepened and the ambiguities that some sentences presented limit the richness of this study. We cannot determine whether the online context promotes more or less self-disclosure. People who are so ashamed may need to participate in order to find a safe place to express themselves; thus, it may be that, despite its limits, online data collection allows for a more diverse set of answers than more established means of qualitative data collection. The lack of participation from trans and non-binary people limits the diversity of our sample and, thus, the extent of results.

### 4.2. Futures Studies

Our results also suggest that the specificities of sexual distressful experiences in LGB+ samples need further investigation to better inform clinicians about possible paths of inquiry, but also to work closely with LGB+ samples to better advocate for the promotion of sexual health and clinical interventions.

Future studies about trans and intersex people’s sexual pleasure are necessary. Although solitary sexual practices are becoming less stigmatized, it is important for future studies to examine the impact of past stigma on current sexual practices.

## 5. Conclusions

The current study sheds light on how LGB+ people may experience specific sexual problems. Diagnostics (such as those in the DSM-5 [4]) do not seem to fit the characteristics of LGB+ people, since most of the presented problems are beyond their sexual function. It also reveals comorbidity among sexual problems within the LGB+ population (part of the study sample perceived as having more than one sexual problem), emphasizing the importance of considering a transdiagnostic approach to treatment as it presents advantages over a categorical approach in addressing comorbid conditions [5,87]. The study participants consider sexual pleasure as a multifaceted phenomenon involving not only the experience but also the interpretation of sexual pleasure. It can serve as a way to explore the self or the partner, but ultimately, its meaning is influenced by the social context in which it occurs [13,22,33,34].

## Figures and Tables

**Table 1:** Frequency of code used for the question “Do you think you have a sexual problem? If yes, could you explain what sexual problem?”.

Codes (*n*)	Excerpts Examples
Lack of sexual desire (30)	*I feel that my sexual partner and I are no longer interested in each other. We have 10 years of relationship.*
Sexual pain (particularly in men) (13)	*Penetration is painful.*
Lack of sexual activity (6)	*I have a sexless marriage.*
Sexual compulsive behavior (5)	*Excessive masturbation.*
Lack of confidence (5)	*Problems related to insecurity.*
Effects of medication/illness (3)	*I take medication for depression and [the medication] is bad for me because I cannot feel pleasure.*
Fear of intimacy (3)	*Fear of the honest and genuine connection that can be provided when having sex with another person.*
Dissatisfaction with relationship configuration (3)	*I feel that I do not get as much pleasure in a monogamous relationship, and I need to feel that I have the freedom to be with someone outside the relationship sporadically even if I don’t actually do it. It creates a feeling of insecurity in my partner, which is understandable.*
Difficulties in being active (gay men) (3)	*Difficulty ejaculating during anal sex (being active).*
Sexual trauma (abuse) (2)	*Sexual abuse trauma.*
Sexual anxiety (2)	*Anxiety during sex.*
Lack of novelty (2)	*Lack of adventure.*
Lack of assertiveness (2)	*Too shy and have difficulty saying “no” when I don’t want to have sex with someone who shows interest.*
Not feeling sexual attraction (2)	*I do not feel sexual attraction [and I feel it is a problem].*
Not having a partner (2)	*Lack of male partner* [answer by a man].
Physical health (Infections) (2)	*Recurrent candidiasis.*
Desire for sex with another gender/sex (1)	*[I am attracted] to someone of the opposite gender to my partner.*

**Table 2:** Frequency of code used for the question “Based on your personal experience, what is solitary sexual pleasure?”.

Category (*n*)	Codes (*n*)
**Enhancing the relationship with oneself** (48)	Self sexual knowledge (20)
	Positive mood inducer (15)
	Promotion of emotional well-being (8)
	Promotion of physical well-being (5)
**Specification of solitary pleasure** (23)	Strictly self-orgasm-oriented (12)
	More exploration of sex toys (4)
	Focus on imagination (4)
	Focus on self-pleasure (3)
**Negative experience** (17)	Lack of fulfillment (8)
	Negative feelings (7)
	Routine (2)
**Unrestrained experience** (15)	Liberation (8)
	Sensorial experience (5)
	Letting oneself go alone (2)
**A goal** (13)	Pleasure even in the absence of another (6)
	A path to partnered sex (5)
	Solitary sex is more pleasurable than partnered sex (2)

**Table 3:** Frequency of code used for the question “Based on your personal experience, what is partnered sexual pleasure?”.

Category (*n*)	Codes (*n*)
**The perks of being with another** (36)	Interconnection of emotions and physical experience (18)
	Honest communication (9)
	Safe place (3)
	Power dynamics (3)
	The pleasure of pleasuring (3)
**Openness to experience** (25)	Letting oneself go with a partner (11)
	Exploration of the 5 senses (7)
	Being in the present moment (7)
**A result of sexual techniques** (16)	BDSM/FET (7)
	Oral sex (5)
	Anal sex (2)
	Finger penetration (1)
	Mutual masturbation (1)
**Psychophysiological experience** (12)	Intense desire/arousal (7)
	Dependent on orgasm (5)
**Misconceptions about sexual pleasure** (10)	It does not depend on orgasm (6)
	It does not depend on penetration (4)
**Absence of intrapersonal constraints** (6)	Physical illness and medication (3)
	Negative states and emotions (3)
**Undesirable feelings** (6)	Difficulty in having pleasure (2)
	Trigger of insecurities (2)
	Waste of time (1)
	Difficulty in surrendering to others (1)
**Explicit Consent** (6)	Consent (6)
**Absence of interpersonal constraints** (2)	Comment about the body (1)
	Comparison with ex-partners (1)

## Data Availability

The data presented in this study can be available on request from the corresponding author. The data are not publicly available due to ethical and privacy restrictions.
